# The Prince of Wales's Fund

**Published:** 1899-02-04

**Authors:** 


### The Institutional Workshop.

### THE PRINCE OF WALES'S FUND.

### A CHAT WITH THE DIRECTOR OF THE NATIONAL HOSPITAL FOR THE PARALYSED AND EPILEPTIC.

When, at the request of the Editors of The Hospital, I asked Mr. Burford Rawlings to see me for a talk about the Prince of Wales's Fund, he replied that he had never yet been interviewed, and had frequently declined invitations of the kind. But after I had explained the scope of the proposed chat, he courteously consented to submit to the ordeal.

"I do so the more readily," he said, "because for many years past I have been wholly in favour of the establishment of a fund such as the Prince of Wales's Fund."

"And your reasons?" I inquired.

"My primary reason is that the managers of a central fund have opportunities for approaching certain individuals and bodies which the managers of individual hospitals do not possess. For instance, I have urged that the life insurance companies should liberally support the hospitals. From 23 to 24 millions sterling are paid annually in premiums in London. But for the work of the hospitals, it is doubtful whether life insurance would be practicable. You must remember that, apart from their treatment of patients, the voluntary hospitals are the source of medical education. For some years the fire insurance companies largely supported the Fire Brigade, and I believe they still continue a measure of support. The life companies could not entertain the application of one hospital; but if a representative organisation like the Prince of Wales's Fund appealed to them they might listen to the appeal."

"Do you think that there are any individuals who might be more amenable to an appeal from a central fund?"

"Yes, in a smaller degree, I believe, great landlords employers of labour, as well as trading companies would give if they knew they would not be pestered fo rhelp in consequence. In present circumstances, if a person contributes to one hospital, the announcement of the subscription usually causes him to be deluged with applications. That is to say, the more liberally a man interprets his obligation the more he is badgered. Under the existing system this cannot be helped, because it is only those who are already donors who respond to appeals."

"This brings us," continued Mr. Rawlings, "to the essence of the Prince's letter, and the now trite statement that in all London there are no more than 50,000 contributors to the hospitals. On the occasion I read a paper before the Hospitals' Association on 'The Chronic Indigence of Our Hospitals,' the Lord Chancellor, who was in the chair, expressed himself as 'appalled' at the statement, and thought it must be erroneous. Subsequently, he verified it. Yet the matter might be put in a more startling manner, for if you were to go through the 50,000 names, you would probably find that a single thousand of the contributors subscribed the greater part of the entire amount. The history of individual hospitals tends to prove the same thing."

"Is it so with yours?"

"Yes, every year a dozen or so of the largest benefactions represent four-fifths of the whole amount contributed. The Prince, in his letter, dwelt upon the small number contributing, and addressed his appeal particularly to the 450,000 families within the metropolitan area in fairly good circumstances who now give nothing. But I fear that most of those who have responded to his appeal are among the regular donors."

"Yet the papers have alluded to the generous response of the public."

"Yes, and I think that the papers make a great mistake in complimenting the public on their generosity to the hospitals. As a matter of fact, the public, by which I mean the overwhelming majority of the population, give nothing at all. It has sometimes occurred to me that if only the people who make direct use of the hospitals for their nominees would subscribe regularly the serious difficulties of the hospitals would vanish, but of course the obligation rests upon all, although upon these more directly."

"What is your experience with regard to such people?"

"Well, we get a large number of country patients—as a 'National' hospital it is our business to receive them. On December 31st we had patients in our wards from 27 different counties, and from large cities like Liverpool, Birmingham, Bristol, Norwich, and York, but we never receive any substantial recognition from these cities. More often than not patients obtain letters from existing subscribers not in their own towns, and many come without letters at all. It is quite common for a well-to-do correspondent to resent the suggestion of a subscription."

"Can you suggest any means by which the Prince of Wales's Fund might be made more successful?"

"My theory is that however big and important a committee is it will not take the place of personal interest and influence. I have known instances in which appeals have been sent out signed by very eminent personages, and the result has been disappointing, whereas the appeal of a humble individual, identified with the particular institution, has been successful. I believe that if some way could be devised of bringing the Prince's vast influence more fully into contact with those who need to be inspired with a measure of his own generous enthusiasm, the success of the Fund would be assured."

"How has the Fund affected your hospital?"

"Beneficially; at least to the extent of the grants made to us, for while I cannot trace the loss of a single subscription to it, our general receipts have been above the average during the last two years."

"What do you think of the manner in which the Fund has been distributed?"

Mr. Rawlings did not answer this question at once, but rejoined:

"I am aware of the dissatisfaction among the smaller hospitals, but although a hospital is not to be condemned because it is small, the managers cannot expect it to be treated as a large institution; and there are small hospitals whose usefulness could be called in question. Moreover, every little hospital, if it is well managed, ought to be able to pay its way without help from the Prince's or any other fund. If you have anything like a case to put forward, it is mere child's play to raise £2,000 or £3,000 a-year; the real difficulty comes when your wants have grown into five figures and upwards. I am lost in surprise as to how the London Hospital, which is spending £70,000 a-year, can get on at all. We are spending £17,000, and I find that is quite enough to be anxious about. As to your leading question," he added, "from the standpoint of individual institutions the actual distribution of the Prince's Fund is perhaps open to criticism, but substantially it seems just."

"In what way is it open to criticism?"

"If you press me to give an example, I might suggest that, possibly, the subscription to Guy's is more liberal than the subscription to the London."

Alfred Wilcox.

